# Effects of wear particles of polyether-ether-ketone and cobalt-chromium-molybdenum on CD4- and CD8-T-cell responses

**DOI:** 10.18632/oncotarget.23757

**Published:** 2017-12-29

**Authors:** Zhe Du, Shujun Wang, Bing Yue, Ying Wang, You Wang

**Affiliations:** ^1^ Department of Bone and Joint Surgery, Renji Hospital, School of Medicine, Shanghai Jiaotong University, Shanghai, China; ^2^ Department of Immunology, Shanghai Institute of Immunology, Shanghai Jiaotong University School of Medicine, Shanghai, China

**Keywords:** joint arthroplasty, wear particles, polyether-ether-ketone, cobalt-chromium-molybdenum, T-cell response

## Abstract

T-cells, second only to macrophages, are often considered as the potential cells involved in debris-related failure of arthroplasty. Here, we assessed the effects of particulate wear debris on T-cells and inflammatory reactions. Blood samples from 25 donors were incubated with polyether-ether-ketone (PEEK) and cobalt-chromium-molybdenum (CoCrMo) particles generated by custom cryo-milling and pulverization. The T-cell phenotypes were assessed using immunostaining and flow cytometry. For the *in vivo* study, 0.1 mL of each particle suspension (approximately 1.0 × 10^8^ wear particles) was injected into murine knee joints; the synovium and spleen were collected one week after the operation for histological examination and immunofluorescence staining. The T-cell responses observed included low-level activation of Th1, Th2, Th17, and CD8+ pathways after 72 h of co-culture of the particles with peripheral blood mononuclear cells. Obvious CD8+ T-cell responses were observed in local synovium and peripheral spleen, with higher inflammatory cytokine expression in the CoCrMo group. Relatively minor cytotoxic and immunological reactions were observed *in vitro*, with PEEK and CoCrMo particle-induced immune responses being primarily mediated by CD8+ T-cells, rather than CD4+ T-cells, *in vivo*. Overall, PEEK wear particles induced fewer inflammatory reactions than CoCrMo particles. This study verified that PEEK was suitable as a potential alternative for metals in total knee replacements in terms of the immunological reaction to PEEK particles, and shed light on the effects of wear particles from polymer and metal-based implants on immune responses.

## INTRODUCTION

Total joint arthroplasty is an established surgical technique that has been successfully used to relieve pain and improve the movement and quality of life of patients with severe joint diseases [1]. Today, total knee replacement (TKR) surgeries are performed worldwide, with excellent results. Artificial knee joints are comprised of cobalt-chromium-molybdenum (CoCrMo) alloys with femoral components articulated using polyethylene (PE) on the tibial surface [2]. Kretzer *et al.* [3] highlights that although the majority of wear arises at the sites of polyethylene, clinically significant amounts of metal wear also occur. In addition, CoCrMo and other metal alloys used in knee components have some further drawbacks. For example, there are concerns regarding potential metal ion release and subsequent osteolysis or allergenicity [4, 5]. In particular, metals used for implantation have a large elastic modulus (approximately 6–20 times greater than that of the surrounding bone) [5–8], causing impaired load force transmission at the implant-tissue interface, stress shielding, and peri-implant bone resorption [5, 9]. Polyether-ether-ketone (PEEK), which exhibits excellent wear-resistance characteristics, mechanical properties, lighter weight, low elastic modulus [5, 10], natural radiolucency, compatibility with magnetic resonance imaging [5, 11, 12], constitutes a potential alternative for metals in metal-on-polymer bearing surfaces. PEEK has become highly attractive for use as a biomaterial for trauma and orthopedic applications, as it has already been successfully employed for spinal surgery [13, 14]. In addition, a recent study revealed the potential of PEEK as a surface material for artificial joints with PE as the other articulating surface [2], and PEEK is used as a component of knee prosthesis (e.g. the bushings and flanges) in current knee products [e.g. the Enduro knee system (Aesculap, Germany)] [14].

Aseptic loosening and peri-prosthetic osteolysis comprise the major complications that limit the long-term survival of prosthetic joints [15]. The mechanisms underlying the development of osteolysis and aseptic loosening are known to influence the longevity of TKRs. Several animal studies have focused on the potential role of wear particles in bone resorption around implants [16]. For example, a study by Pearson *et al.* had demonstrated that CD4+ T-cells were activated in response to wear debris from CoCrMo alloys [17]. Previous animal studies have shown that metal hypersensitivity was mediated by T-cells in classical delayed-type hypersensitivity (DTH)-type responses (DTH type IV). The effector phase of a DTH response is initiated by a contact between sensitized T-cells and an antigen presented in a class-II major histocompatibility complex (MHC) by antigen-presenting cells (APCs). In this phase, T-cells, which are activated by antigens, are characterized as helper T-cells, which, in conjunction with APCs, can secrete a variety of cytokines that recruit and activate macrophages, monocytes, neutrophils, and other inflammatory cells [18, 19]. Previous studies have shown an increased proportion of CD8+ T-cells and a decreased ratio of CD4+ to CD8+ T-cells in the peripheral blood during revision arthroplasty, compared with the blood during primary arthroplasty [20]. Additionally, histological examinations revealed that metal wear induced a typical non-specific inflammatory response as well as a specific metal hypersensitivity immune reaction [21].

However, to assess the feasibility of its use as a potential alternative for metals in TKRs, the mechanisms by which the immune system reacts to PEEK particles need to be determined. Previous studies have made some headway in understanding the role of macrophages and the cytokine secretion profiles in response to PEEK debris. For example, Hallab *et al.* found that there were no significant differences between the effects of PEEK and PE particles on macrophage viability or proliferation [22]. In addition, subtle elevations in inflammatory cytokines indicated a mild persistence of responses to PEEK debris in an animal model [23]; however, it is also unknown whether the immune response to PEEK particles is stronger than that to metals. We hypothesized that, similar to CoCrMo particles, the proliferation reactivity (peripheral blood mononuclear cells [PBMCs] or local tissues) responses to challenges with PEEK particles would also be mediated by both macrophages and early T-cell activation. In this study, we aimed to determine the effect of particulate wear debris on the immune system, with a focus on T-lymphocytes (both CD4+ and CD8+ T-cells), and to identify the effects of two types of wear particles on cytotoxic and immunological reactions.

## RESULTS

### Wear particle characterization

**Figure 1:**
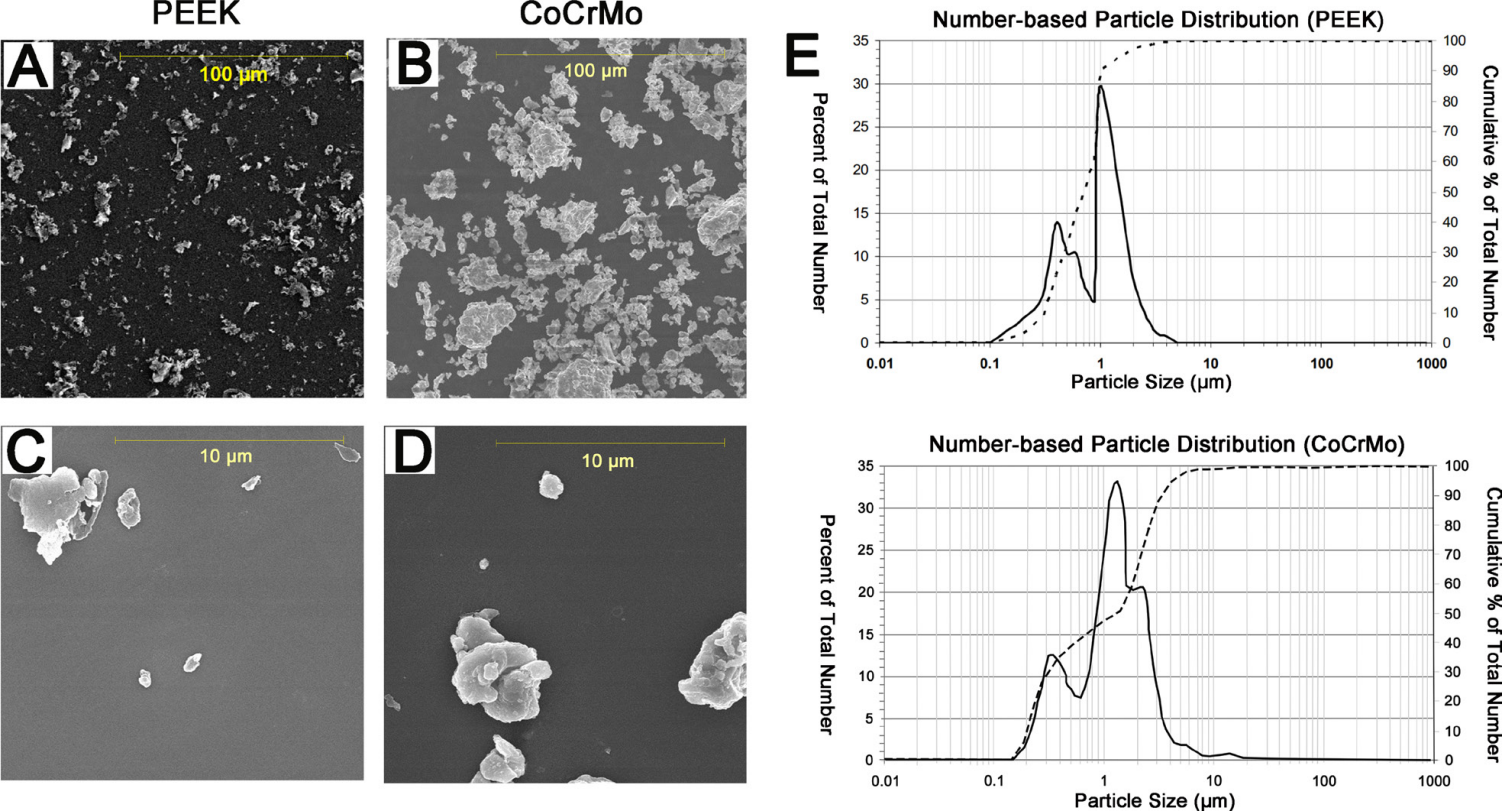
Scanning electron microscopy (SEM) images and size distribution (number-based) of wear debris from polyether-ether-ketone (PEEK) and cobalt-chromium-molybdenum (CoCrMo) groups (**A** and **C**) SEM images of PEEK wear debris in two magnification fields (A. magnification 1000×, C. magnification 10,000×). (**B** and **D**) SEM images of CoCrMo wear debris in two magnification fields (B. magnification 1000×, D. magnification 10,000×). (**E**) Size distribution of PEEK and CoCrMo particles, as determined using SEM.

The scanning electron microscope (SEM) micrographs showed that the particles had similar morphological characteristics; i.e., globular or granular shapes, at all size ranges (Figure 1A–1D). The mean particle diameter of the PEEK samples was 1.05 μm. The range was 0.25–46.47 μm, with 99% of the particles measuring less than 5 μm and 93% in the submicron range (Figure 1E, Table 1). The mean diameter of the CoCrMo particles was 3.16 μm. The range was 0.39–61.65 μm, with 90% of the particles measuring less than 5 μm and 50% in the submicron range (Figure 1E, Table 1). The area, perimeter, ellipse major axis, and ellipse minor axis measurements are shown in Table 1.

**Table 1:** SEM particle analysis of PEEK and CoCrMo particles

	PEEK	CoCrMo
Particle size (μm)	1.05 (0.70)	3.16 (2.13)
Aspect ratio	1.83 (1.53)	1.81 (1.59)
Roundness	0.61 (0.52)	0.60 (0.51)
Form factor	0.67 (0.60)	0.67 (0.60)
Perimeter (μm)	4.51 (2.12)	5.96 (3.65)

Parameter values are reported as means (medians).

### Effects of wear particles on T-cell populations

**Figure 2:**
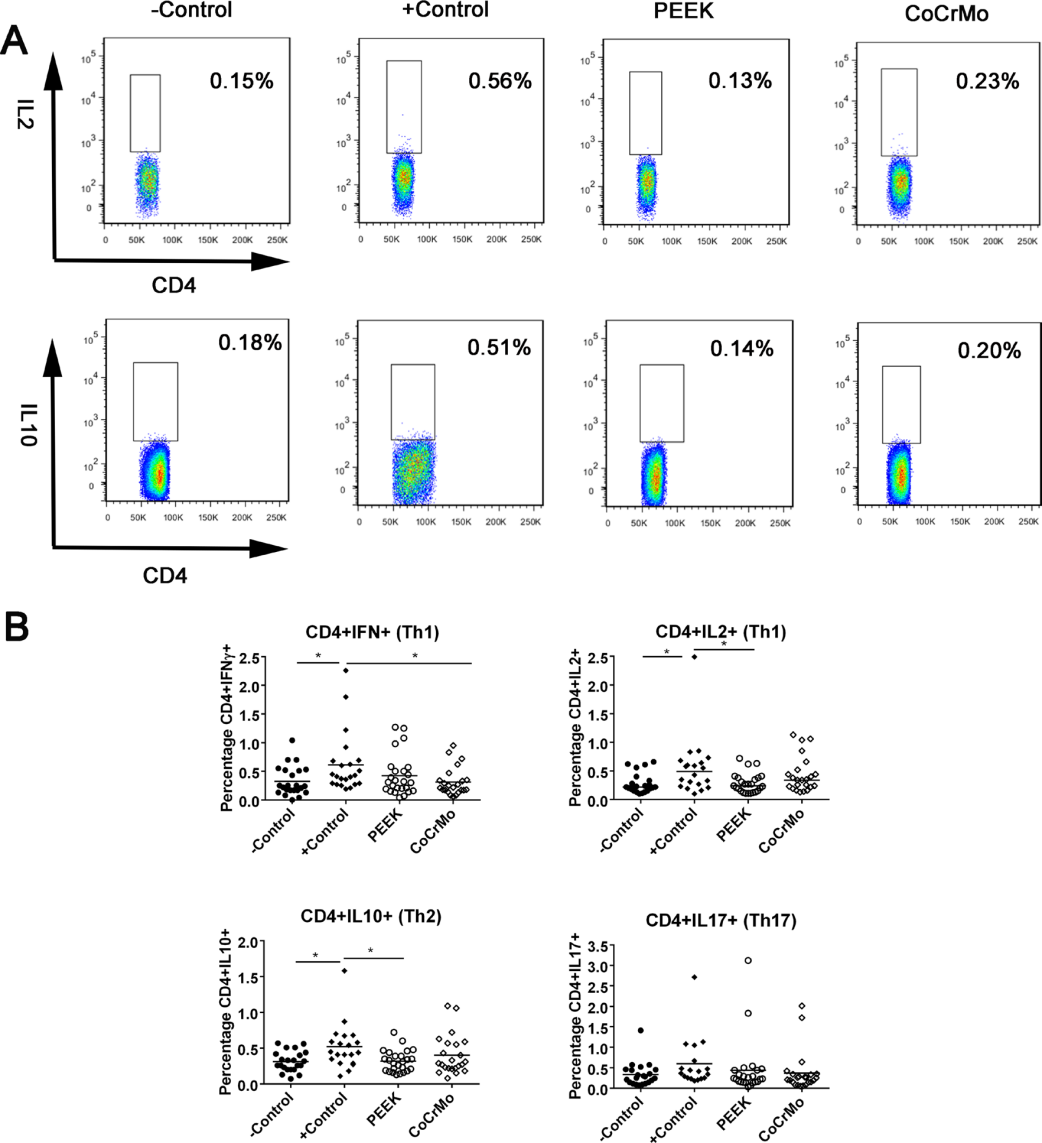
CD4+ T-cell populations identified by immunostaining and flow cytometry in human peripheral blood (**A**) Raw data showing differences between PEEK and positive control groups in the induction of CD4+IL2+ and CD4+IL10+ T-cell responses. (**B)** Th1, Th2, and Th17 cells are shown in the control group or the groups challenged with PEEK, and CoCrMo particles. The −Control group was unchallenged, resuspended in RPMI 1640 medium; the +Control group was treated with human T-activator CD3/28. Each data point represents the response of an individual participant; the bar indicates the mean value. ^*^P < 0.05.

The full gating strategies are shown in Supplementary Figure 1 (taking the identification of CD4+IL2+ T-cell populations induced by CoCrMo wear particles as an example). For CD4+ T-cell responses in human peripheral blood, there were no significant differences between the experimental groups (PEEK and CoCrMo) and the negative control group. Compared to the positive control, the experimental groups all showed decreased or similar responses. For example, PEEK debris showed lower responses than the positive control in CD4+IL2+ (*p* = 0.008) and CD4+IL10+ (*p* = 0.003) cells. There was no difference between PEEK and CoCrMo wear particles in CD4+ T-cell responses (Figure 2).

**Figure 3:**
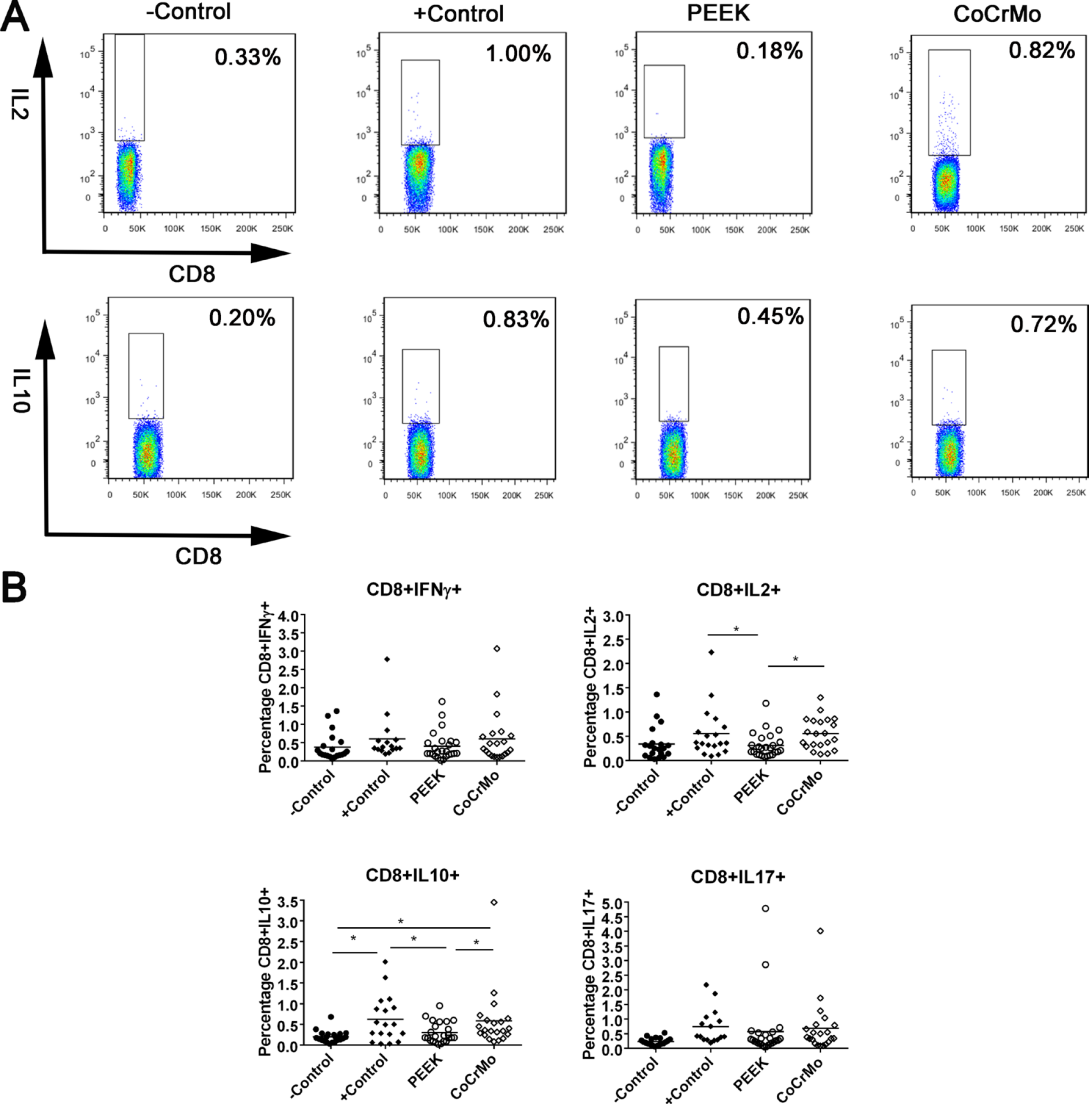
CD8+ T-cell populations identified by immunostaining and flow cytometry in human peripheral blood (**A**) Raw data showing differences between PEEK and CoCrMo particles in the induction of CD8+IL2+ and CD8+IL10+ T-cell responses. (**B**) CD8+IFNγ+/IL-2+/IL-10+/IL-17+ cells in the control groups or the groups challenged with PEEK, and CoCrMo particles. The −Control group was unchallenged, resuspended in RPMI 1640 medium; the +Control group was treated with human T-activator CD3/28. Each data point represents the response of an individual subject; the bar indicates the mean value. ^*^P < 0.05.

For CD8+ T-cell responses in human peripheral blood, there were no significant differences between the experimental groups (PEEK and CoCrMo) and the negative control group, except for CoCrMo, which exhibited a higher CD8+IL10+ T-cell response than the negative control (*p* = 0.006; Figure 3). Compared to the positive control, the experimental groups all showed decreased or similar responses. PEEK debris showed lower responses than the positive control in CD8+IL2+ (*p* = 0.027; Figure 3) and CD8+IL10+ T-cell responses (*p* = 0.021; Figure 3). Clear differences were observed between PEEK and CoCrMo particles in the induction of CD8+IL2+ and CD8+IL10+ T-cell responses, with lower responses in PEEK debris (*p* = 0.023 and 0.036, respectively; Figure 3).

### Histological analysis of the rat synovium

**Figure 4:**
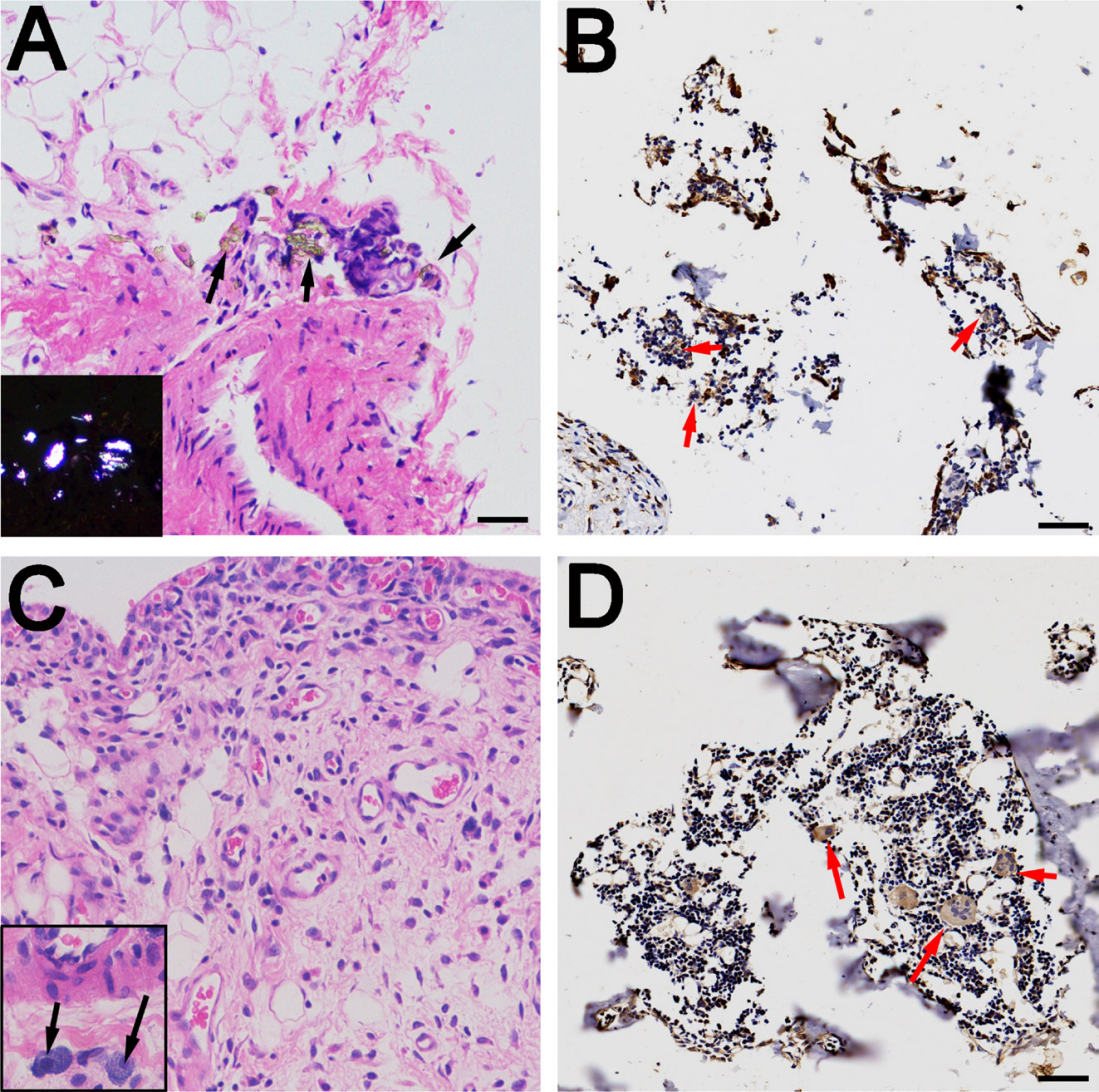
Histological examination of rat synovium tissues (**A** and **C**) Hematoxylin and eosin staining of synovium (A for PEEK, C for CoCrMo), indicated by black arrows; wear particles (A) and inflammatory cells (C) can be observed in the necrotic tissues. Polarizing microscopy of PEEK particles is shown in the lower left quarter. (**B** and **D**) Immunohistochemical staining of synovium (B for PEEK, D for CoCrMo), indicated by red arrows; multinucleated giant cells and Langhans giant cells are concentrated at the sites of wear particles. Bar = 50 μm.

**Figure 5:**
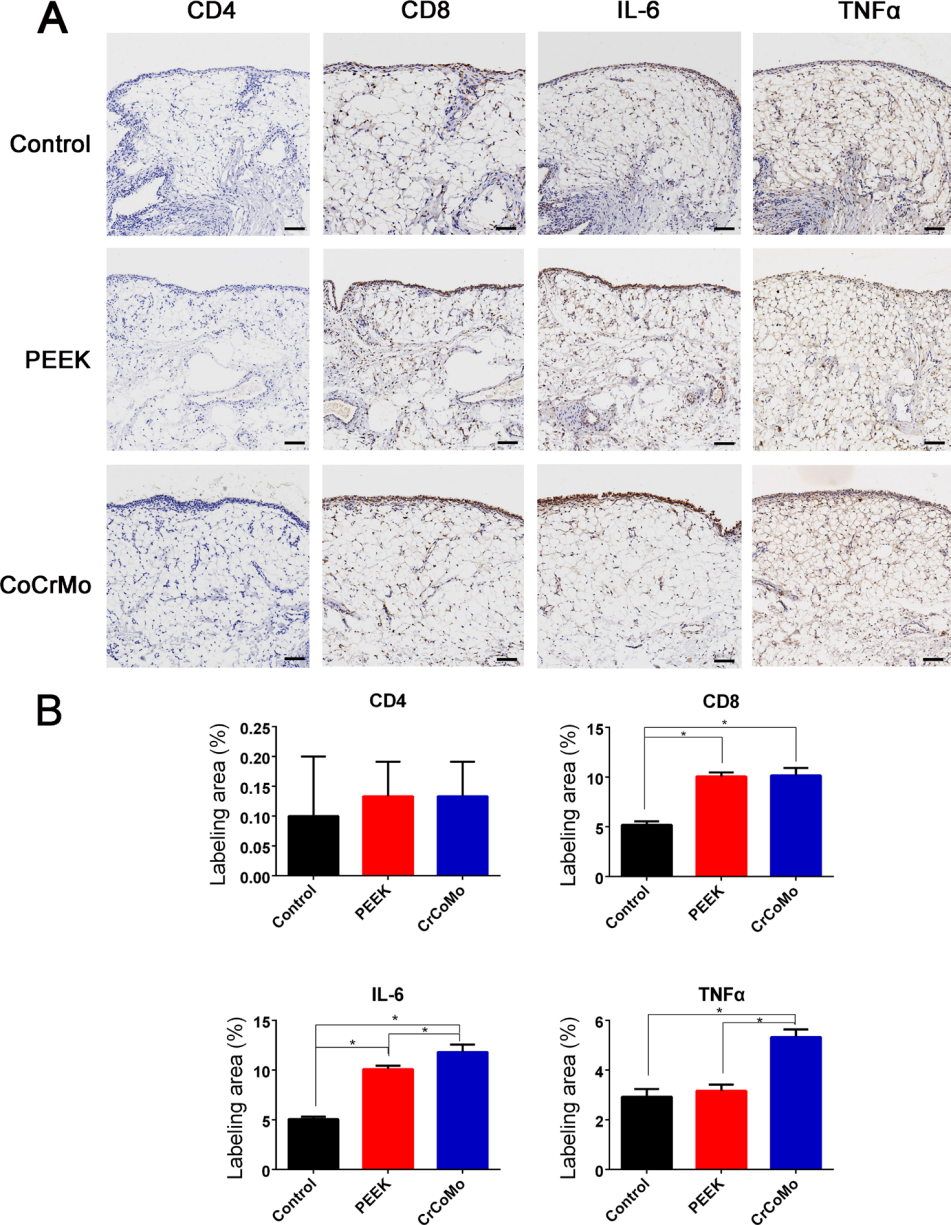
Immunohistochemical staining and analysis of synovium (**A**) Immunohistochemical staining (CD4, CD8, IL-6, and TNFα) of synovium in three groups. (**B**) Semiquantitative analysis of the positive area. Bar = 100 μm. ^*^P < 0.05.

The detection of wear particles is shown in Figure 4A–4D. Multinucleated giant cells were concentrated in particle-containing areas (Figure 4B and 4D). In the immunohistochemical analysis, almost no CD4+ T-cells were found in the control or experimental groups (Figure 5). However, CD8+ T-cells were highly expressed in experimental groups, with comparable expressions in the PEEK and CoCrMo groups (Figure 5). Inflammatory factors (IL-6 and TNFα) showed increased expression in the CoCrMo groups (Figure 5).

### Immunofluorescence labeling of the rat spleen

**Figure 6:**
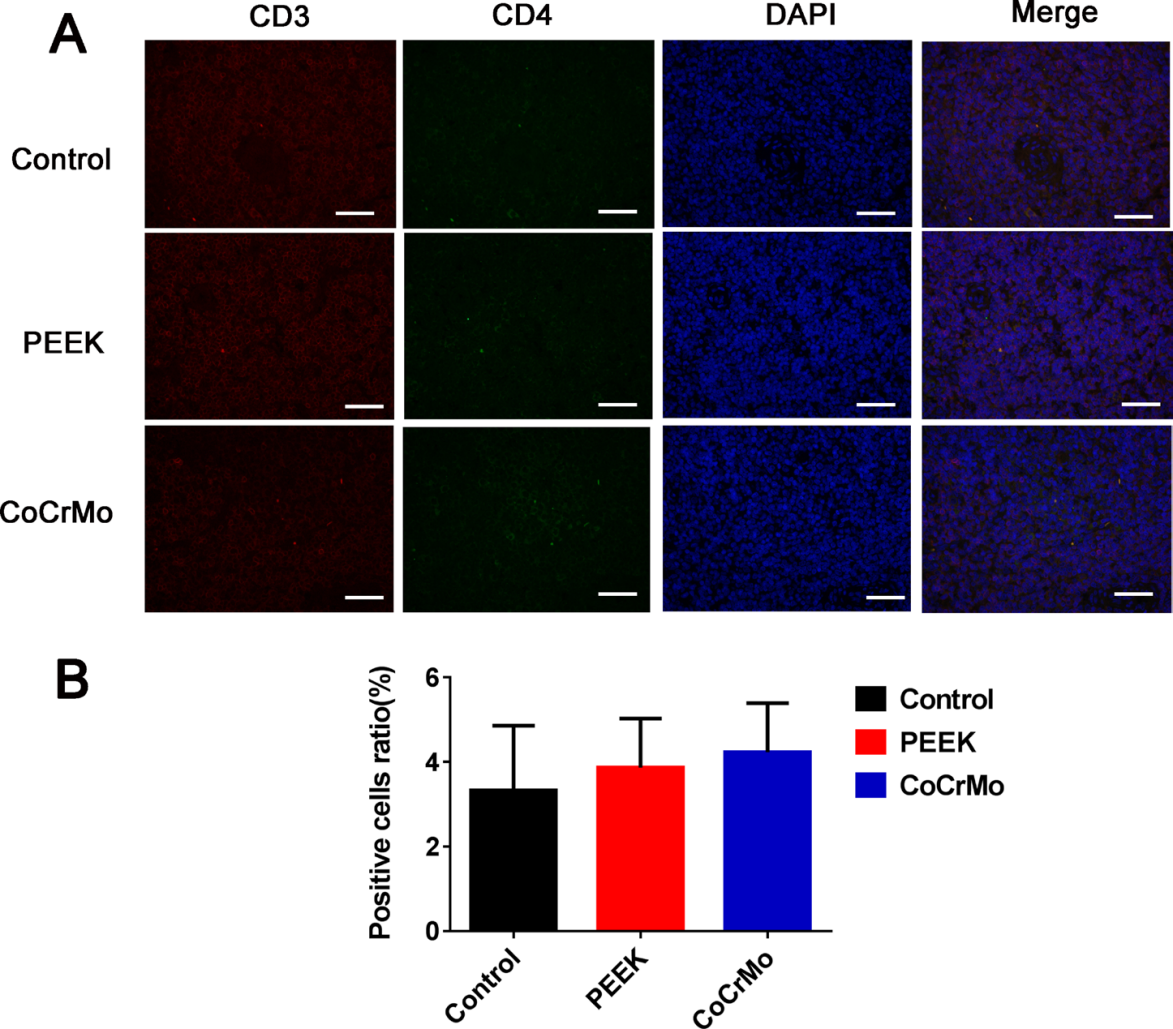
Immunofluorescence labeling of CD4+ T-cell responses (**A**) Immunofluorescence labeling of CD4+ T-cells of the spleen in three groups. (**B**) Analysis of the ratio of positive cells. Bar = 50 μm.

**Figure 7:**
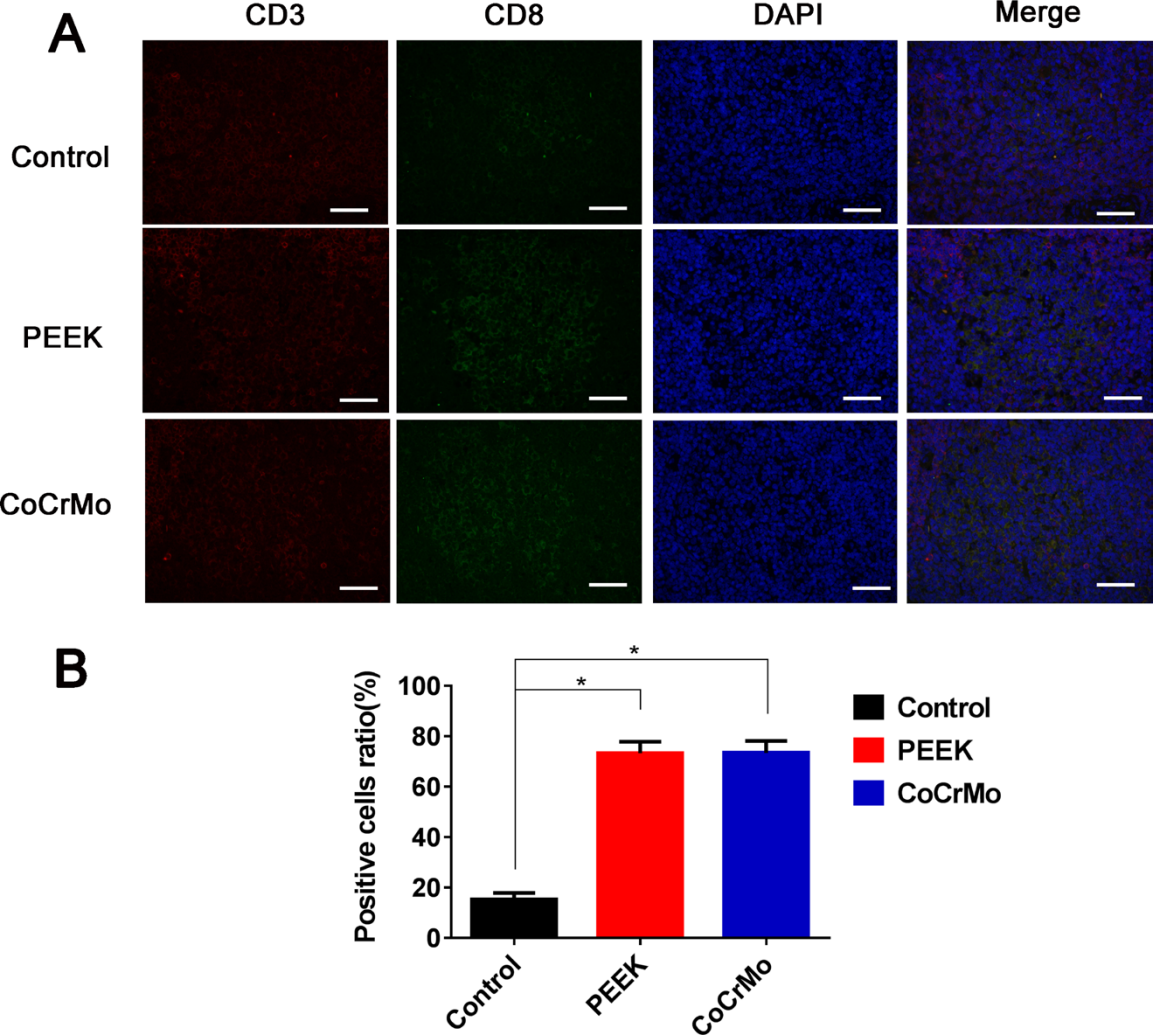
Immunofluorescence labeling of CD8+ T-cell responses (**A**) Immunofluorescence labeling of CD8+ T-cells of the spleen in three groups. (**B**) Analysis of the ratio of positive cells. Bar = 50 μm. ^*^P < 0.05.

With respect to CD4+ T-cell responses in the rat spleen, there were no significant differences between the experimental groups (PEEK and CoCrMo) and the negative control group (Figure 6). In contrast, for CD8+ T-cell responses, an increased number of positive cells were found in the PEEK and CoCrMo groups compared to controls, with comparable responses between the two groups (Figure 7).

## DISCUSSION

In this study, we assessed the effect of particulate wear debris on T-cells. We also investigated the effects of two kinds of wear particles on cytotoxic and immunological reactions. Our findings demonstrated that PEEK and CoCrMo particles induced immune responses that were mainly mediated by CD8+ T-cells, and that PEEK wear particles induced fewer inflammatory reactions than CoCrMo particles, providing important insights into the immunological reactions caused by these common wear particles (Supplementary Figure 2).

The wear particles from bearing materials can affect the longevity of total joint arthroplasty, as debris can elicit biological responses and cause periprosthetic osteolysis, leading to loosening of the prosthesis [16]. Numerous factors are thought to affect the cellular response to wear particles including particle composition, dose, volume, size, and shape [24–26]. Particles in the phagocytosable size range (0.1–10 μm in diameter) are considered the most biologically reactive, particularly those with a mean size of 1 μm [27]. Green *et al.* [28] showed that 0.45- and 1.71-μm-sized PE particles were necessary to stimulate cytokine release and bone resorbing activities. In the present study, the size of the wear particles was in the range of 0.1–102 μm in diameter, with an average diameter of 1–3 μm, which was thus biologically phagocytic and phlogistic.

Lymphocyte activation requires the simultaneous delivery of an antigen-specific signal associated with MHC proteins and a “costimulatory” signal by APCs [29, 30]. When PBMCs treated with debris for over 72 h were initially processed by macrophages, downstream activation of T-cells was observed. Previous clinical and *in vitro* studies have shown that metal hypersensitivity is mediated by T-cells in DTH type-IV responses [19]. Previous studies have also examined the specific roles of Th1 and Th2 lymphocytes in osteolysis and aseptic loosening in TKRs [30]. In particular, the Th1 response is crucial for the activation of macrophages and cytotoxic T-lymphocytes, and is known to be involved in cell-mediated immune responses. In comparison, the Th2 response is the most effective activator of B-lymphocytes and is associated with humoral immunity [30, 31]. Th17 cells play an important role in the initiation of inflammation by recruiting neutrophils and macrophages to sites of infection or sites of tissue damage, in the case of sterile inflammation [17, 32]. However, in contrast with previous literature, in both *in vitro* and *in vivo* studies, we verified that the two types of particles induced relatively low levels of CD4+ T-cells, especially in synovial tissues and peripheral spleen. Similarly, Willert *et al.* [33] had reported that metal particle-induced responses were not characteristic of a delayed-type IV hypersensitivity reaction, but rather comprised aseptic lymphocyte-dominated vasculitis-associated lesions (ALVAL) or lymphocyte-dominated immunological answers (LYDIA).

In *in vitro* studies, CD8+ T-cells were also induced in relatively low levels, with PEEK particles showing lower responses in the induction of CD8+IL2+/IL10+ T-cells. However, in *in vivo* studies, CD8+ T-cells were more highly induced in local synovial tissues and peripheral organs than the control group. In accordance with this, previous studies have shown an increased proportion of CD8+ T-cells and a decreased ratio of CD4+ to CD8+ T-cells in the peripheral blood lymphocytes of patients with worn implants [20]. Furthermore, Hailer *et al*. [34] had reported a correlation between the percentage of CD8+ T-cells and the concentration of chromium and cobalt in the blood of patients. The mechanisms behind this phenomenon were unclear, and it was speculated that CD8+ T-cells were specific for class-I MHC molecules on foreign proteins [35, 36]. In the present study, the wear particles were able to induce apoptosis and/or direct necrosis of endothelial cells, causing the accumulation of macrophages and Langerhans cells (shown in Figure 4). These necrotic substances and APCs might activate the class-I MHC molecular pathway, resulting in elevated CD8+ T-cell response. The apoptosis of T-lymphocytes may also be responsible for imbalanced CD4+/CD8+ T-cell responses. For example, Stefan *et al.* [36] reported that the number of CD4+ cells decreased during strong apoptotic reactions, whereas CD8+ cells were affected to a much lesser degree. Thus, the high incidence of CD8+-related lymphocyte reactivity and the subsequent *in vivo* activation may contribute to the etiology of debris-induced osteolysis.

Previous studies have reported that metallic debris derived from alloy implants induced macrophage activation and triggered immune responses, releasing an array of pro-inflammatory mediators, including TNF-α, IL-1, IL-6 [37, 38], and colony-stimulating factors such as macrophage- and granulocyte-macrophage colony-stimulating factor [38, 39]. In this study, we examined the levels of inflammatory factors (IL-6 and TNFα) in the local tissues, and found that CoCrMo particles induced more inflammatory factors than PEEK particles, and that the inflammatory factors were induced not only by macrophage activation, but also by T-cell activation. This is consistent with a proposal by Bainbridge *et al.* [40, 41], that intracellular particles from the prosthesis, along with elevated costimulatory molecule expression, might promote T-cell inflammatory reactions in the prosthetic tissues.

There were several limitations to this study. First, the wear condition alone may not represent the nature of the wear in clinical cases; thus, further studies using wear particles isolated from tissues or validated joint replacement simulators are required, instead of manufactured particles. Second, although metal ions have been reported to be a theoretical source of genotoxicity [42], we did not investigate the immunogenicity of degradation products of CoCrMo or PEEK particles. Third, it remains unknown whether the complex environment of the peri-implant milieu ultimately mitigates or enhances cell susceptibility to the effects of particle exposure *in vivo*. Thus, the results of this study established a foundation for more sophisticated studies using mixed cell populations or direct *in vivo* osteolysis models.

In summary, relatively minor cytotoxic and immunological reactions were observed *in vitro*, whereas PEEK and CoCrMo particle-induced immune responses were mainly mediated by CD8+ T-cells, rather than CD4+ T-cells, *in vivo*. PEEK wear particles induced fewer inflammatory reactions than CoCrMo particles, suggesting that PEEK was suitable as a potential alternative for metals in TKRs in terms of the relative immunological reaction to PEEK particles. Overall, the results of this study shed light on the effects of wear particles from polymer and metal-based implants on immune responses and demonstrate that determining the mechanism of T-cell activation may be important for developing therapeutic tools against aseptic loosening induced by PEEK and CoCrMo particles. Future studies should focus on the specific mechanisms of CD4 and CD8 lymphocyte-mediated osteolysis.

## MATERIALS AND METHODS

### Participants

The study included 25 participants (12 men and 13 women; average age: 62.1 years [range, 40–71 years] and 65.7 years [range, 54–75 years], respectively). Patients with knee osteoarthritis were chosen, as only they would need knee prosthesis, and their blood would be exposed to wear debris. Patients with known inflammatory diseases and recent infections were excluded. All participants provided written informed consent, and ethical approval for the protocols was received from the Renji Hospital, Shanghai Jiaotong University School of Medicine (grant no. 2017-021).

### Generation of wear particles

Commercially available particles (BioEngineering Solutions, Oak Park, IL, USA) were generated from non-sterilized bulk material of a PEEK tibial tray and an highly cross-linked polyethylene (HXLPE) insert (Zeniva PEEK ZA-500, Chirulen HXLPE 1020X; Jiangsu Okani Medical Technology Co., Ltd., Soochow, JS, China), using proprietary techniques involving custom cryo-milling and pulverization. The CoCrMo wear debris was a gift from 3D Systems, Inc. (Rock Hill, SC, USA). The debris were isolated in ethanol and progressively filtered through polycarbonate membranes. Endotoxins were removed using Pyroclean. The debris were washed thrice in 70% ethanol, dried from a stock solution of more than 2 mg/100 μL of ethanol, and then tested for endotoxins. The particles were subjected to ethylene oxide (EtO) sterilization. Particles were then resuspended in ethanol and re-dried in a vacuum oven under sterile conditions.

### Characterization of wear particles

Particle size analysis was performed using an SEM (PSEMII Aspex, Pittsburg, PA, USA) with a liquid sample circulator and an ultrasound dispersal system. The system was flushed 10 times with fresh reverse-osmosis-deionized water (> 18 MOhm) and sterile-filtered (0.2-μm pores) to remove potential contaminants. The particle-DH_2_O solution was added to the liquid circulator at 1 mL/s, to obtain the required laser obscuration value of less than 3%, as indicated by the automated software. The measurement integration time was 15 s, with three repeat measurements for each sample. Next, 100–200 μL from the remaining 1–2 mL of enzyme/acid-processed sample was vacuum-dried on a polycarbonate membrane (0.1–0.01-μm pores, GTTP; Thermo Fisher Scientific, Waltham, MA, USA). All the membranes were dried in a desiccator at 22°C for 24 h, prior to SEM analysis.

The total number of particles, regardless of size, was counted in at least 15 random image fields on each filter. The sizes and shapes of the particles were assessed by image analysis of the micrographs (Scion Image/NIH Image analysis software). The minimum number of particles in each sample was 450. The software program, Scion Image, was used to find the following measurements for each particle on the image: area, perimeter, ellipse major axis, and ellipse minor axis (for defining the particle dimensional measurements).

### Challenging PBMCs with debris

A total of 20 mL of peripheral blood, with approximately 5 × 10^6^ PBMCs, was isolated from each participant and subjected to density gradient centrifugation. The PBMCs were separated into five groups (two control groups and three experimental groups), and wear debris was added at a concentration of 20 particles/PBMC [17]. The negative control was unchallenged, being resuspended in RPMI 1640 medium (Thermo Fisher Scientific), and the positive control group was treated with human T-activator CD3/28 (Thermo Fisher Scientific), which is known to activate human T-cells. The PBMCs were incubated at 37°C and 5% CO_2_ for 72 h, and then analyzed by flow cytometry as described below.

### T-cell phenotype and cytokine secretion

The T-cell populations and cytokine secretion were analyzed by immunostaining and flow cytometry, using fluorochrome-conjugated antibodies against CD3 (PB), CD8 (PerCP), interleukin (IL)-10 (PeCy7), IL-17 (PE), IL-2 (APC), and interferon (IFN)-γ (FITC) to identify Th1, Th2, Th17, and CD8+ T-cells. An anti-CD4 antibody was not used; CD3 positive and CD8 negative cell populations were identified as CD4+ T-cells in flow cytometry analyses (Supplementary Figure 1). All the antibodies were obtained from eBioscience (Thermo Fisher Scientific). Fluorescence staining was detected using a CyAn ADP flow cytometer (Beckton Dickinson) and analyzed using GraphPad Prism 5 software (La Jolla, CA, USA).

### Animals

All the animal procedures and experiments were approved by the Animal Ethical Committee of the Renji Hospital, Shanghai Jiaotong University, School of Medicine (Shanghai, China). We obtained 30 female Sprague-Dawley rats, weighing 200–250 g. The rats were kept in a surgical research institution for one week prior to particle injection. They were kept in groups of three per cage and allowed food and water ad libitum. They were randomly assigned to one of three treatment groups: control (*n* = 10), PEEK particles (*n* = 10), and CoCrMo particles (*n* = 10).

### Surgical procedures

Briefly, the rats were anesthetized by intraperitoneal injection of ketamine (10 mg/kg). Each rat was immobilized with the knee joint in the maximally flexed position, and the right leg was shaved and depilated. The two types of particle suspensions were sonicated for at least 60 min to avoid particle aggregation prior to injection. Each particle suspension (0.1 mL; approximately 1.0 × 10^8^ wear particles) was then injected into the right knee of the experimental groups under sterile conditions. PBS (0.1 mL) was injected into the right knee of the control group. After surgery, the rats were housed in ventilated rooms with access to water and food.

### Sample preparation

The rats were sacrificed one week post-surgery. The synovial tissues in the knee joints and spleen tissues were harvested and fixed in 4% buffered formaldehyde for histomorphometric observation and immunofluorescence labeling. The labeled area in the immunohistochemistry procedure was analyzed using Image-pro plus 6.0 (Media Cybernetics, Inc., Rockville, MD, USA). A total of six fields, at 200× original magnification of each slice, were digitized and transferred to the Image-pro plus 6.0 software. The area covered by positive cells (brown color) was determined, and the brown-labeled area was then divided by the area of cells and multiplied by 100.

### Immunohistochemistry

For each knee synovial membrane, two sections were stained immunohistochemically with each primary antibody (CD4, CD8, IL-6, and TNF-α [R&D Systems, Minneapolis, MN, USA]). Additionally, for one section of each knee, PBS was used instead of the primary antibody, serving as negative control. After staining, the two samples of each primary antibody were evaluated semiquantitatively with a light microscope using different magnifications (10×, 20×; Carl Zeiss MicroImaging GmbH, Oberkochen, Germany) [43].

### Double immunofluorescence staining of CD4+ and CD8+ T-cells in the spleen

The primary antibodies comprised a mixture of two antibodies (CD3 [rabbit antibodies] +CD4/CD8 [mouse antibodies]). For secondary antibodies, mixtures of Alexa Fluor Cy3-conjugated goat-anti-mouse IgG and Alexa Fluor 488-conjugated goat-anti rabbit IgG were used. Cells that showed double staining in the immunofluorescence procedure were manually counted in six fields at 1000× original magnification. The number of double-stained cells was then divided by the total number of cells and multiplied by 100 [44].

### Statistical analysis

Data were analyzed using one-way analysis of variance (ANOVA) with LSD post-hoc *t*-tests. Mann-Whitney U or Wilcoxon rank sum tests were used for unpaired and paired nonparametric data. Differences with *P* < 0.05 were considered statistically significant.

## SUPPLEMENTARY MATERIALS FIGURES



## Abbreviations

PEEK: Polyether-ether-ketone
CoCrMo: Cobalt-chromium-molybdenum
TKR: Total knee replacement
PE: Polyethylene
MHC: Major histocompatibility complex
APCs: Antigen-presenting cells
PBMCs: Peripheral blood mononuclear cells
SEM: Scanning electron microscope
