# Supplementary material for: Effects of wear particles of polyether-ether-ketone and cobalt-chromium-molybdenum on CD4- and CD8-T-cell responses
Source: Oncotarget. 2017 Dec 29;9(13):11197–208. doi: 10.18632/oncotarget.23757 (PMC5834256; doi:10.18632/oncotarget.23757)
Supplement: Supplementary file 1 [file oncotarget-09-11197-s001.pdf]

Effects of wear particles of polyether-ether-ketone and cobalt-chromium-molybdenum on CD4- and CD8-T-cell responses

SUPPLEMENTARY MATERIALS

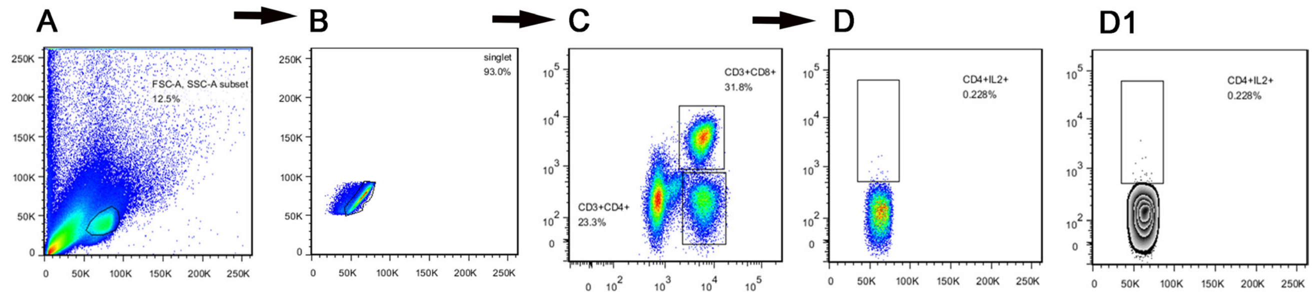

Supplementary Figure 1: The full gating strategies used in identifying CD4+IL2+ T-cells populations induced by CoCrMo wear particles. (**A**) Lymphocyte subsets were delineated. (**B**) Adherent lymphocytes were removed. (**C**) CD4+ lymphocyte subsets and CD8+ lymphocyte subsets were respectively delineated. (**D**) Percentage of lymphocytes secreting IL-2 in CD4+ lymphocyte subsets was delineated (Pseudocolor). (**D1**) Percentage of lymphocytes secreting IL-2 in CD4+ lymphocyte subsets was delineated (Zebra).

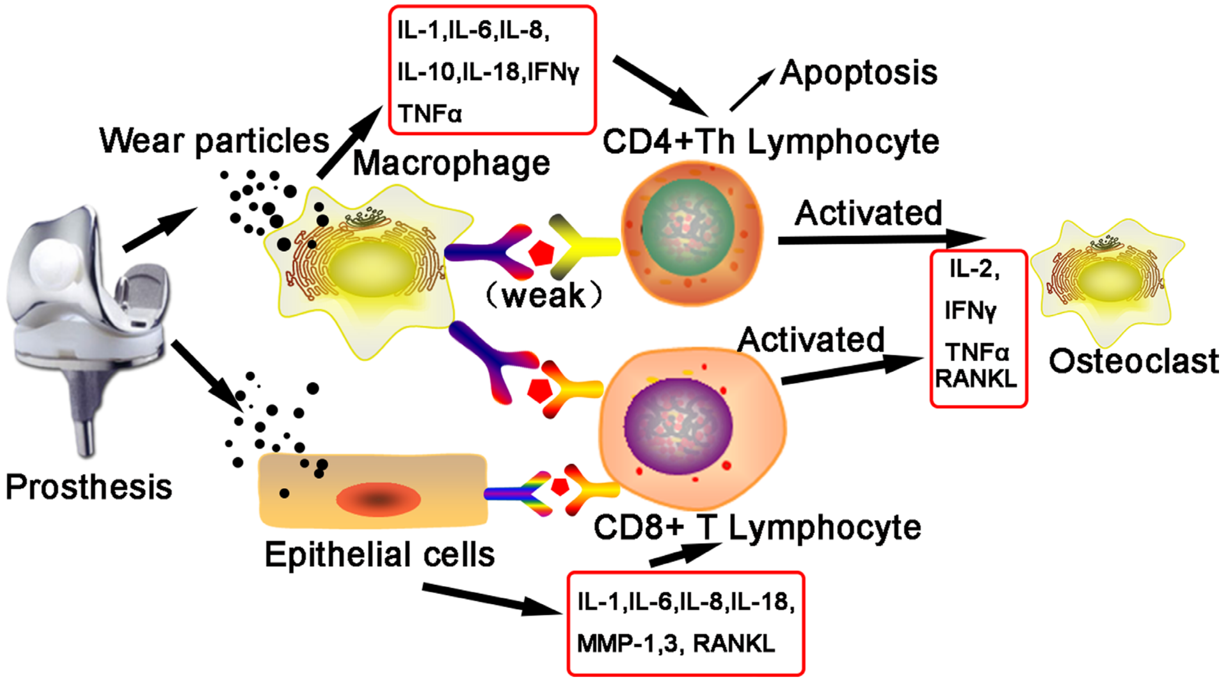

Supplementary Figure 2: A schematic illustration of the proposed mechanism depicting the effects of wear particles from polymer and metal-based implants on immune responses.
